# Influence of the cautionary legend on non-nutritive sweetener (NNS) on preference and healthfulness perception

**DOI:** 10.1371/journal.pone.0314040

**Published:** 2024-11-25

**Authors:** Claudia Calderon, Tania C. Aburto, Carolina Batis, Alejandra Contreras-Manzano, Simón Barquera

**Affiliations:** 1 Gillings School of Global Public Health, University of North Carolina at Chapel Hill, Chapel Hill, North Carolina, United States of America; 2 National Institute of Public Health, Center for Research on Nutrition and Health, Cuernavaca, Morelos, Mexico; 3 National Council of Humanities, Sciences and Technologies, Mexico City, Mexico; Institute of Apicultural Research, CHINA

## Abstract

In 2020, Mexico’s Congress mandated front-of-package warning labels (FOPWL) and two cautionary legends; one of which for non-nutritive sweeteners (NNS) with a statement “Contains NNS. Avoid in children”. The aim of the study was to assess the influence of the “excess in sugar” warning label (WL) and NNS cautionary legend on preference and healthfulness perception of fruit-based beverages among parents of 5–10 year-olds. Also, to test if parents’ preferences and perceptions differed by nutrition knowledge and previous knowledge on NNS. Data from the EPHA niñ@s (Study of the Perception and Dietary Habits in Children, for its acronym in Spanish) cohort were analyzed (n = 844). Parents were asked to choose between 100% juice and nectar with added sugars above the cut-off point, and between the latter and nectar with NNS marketed as “light”, and to rate how healthy they considered each product at two different timepoints. At time 1, products were shown without the FOPWL; at time 2, nectar with excess sugar had the “excess sugar” WL and nectar with NNS had the cautionary legend on NNS. General Linearized Models (GLM) were used to assess changes in preference and perceived healthiness. Interaction terms assessed the impact of nutrition knowledge and NNS awareness. The study found that FOPWL significantly shifted parental preferences towards 100% juice over nectar with “excess sugar” (15.4% increase, p<0.001), and the latter over nectar with NNS (21.8% increase, p<0.001). Similarly, the FOPWL decreased the perceived healthiness of both nectar with “excess sugars” and nectar with NNS. The effect of labels on healthfulness perception was strongest among parents with low to medium nutrition knowledge and no prior knowledge of NNS. The inclusion of FOPWL seems to aid parents in making better-informed decisions regarding the nutritional quality of beverages for their children.

## Introduction

The prevalence of obesity among adults in Latin America and the Caribbean has tripled since 1975 and has since driven the burden of non-communicable diseases (NCDs) for this region [1]. A pervasive contributing factor to the obesity epidemic is the increased consumption of ultra-processed foods and beverages [2]. In response, some countries in Latin America have incorporated policy-level interventions through the implementation of front-of-package warning labels (FOPWLs) in pre-packaged products. FOPWLs were first implemented in Chile followed by Peru, Uruguay, and Mexico that introduced similar warning label systems [3].

**Fig 1:**
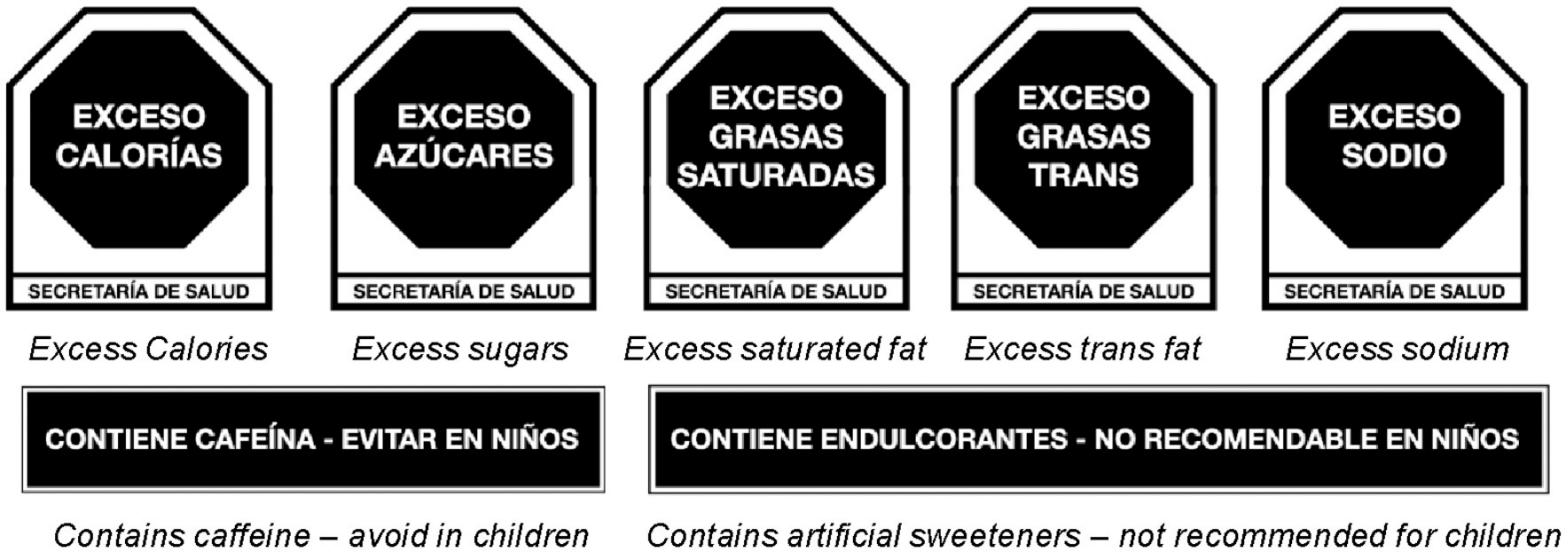
Mexican warning label system.

In Mexico, FOPWLs were implemented in October 2020. These labels consist of five black octagons on pre-packaged products, indicating excessive content of calories, added sugars, sodium, saturated fat, and trans-fat [3]. These warning labels follow the cut-off points established by the Pan American Health Organization (PAHO) nutrient profile model and to the Chilean criteria for excessive calorie content [4,5]. Additionally Mexican FOPWLs system includes innovative precautionary legends for caffeine and non-nutritive sweeteners (NNS) with a statement “avoid in children” and “not recommended for children”, respectively [3] (Fig 1).

In general, FOPWL serve as a public health policy aimed at upholding the right to accessible, simple, and truthful information for consumers to encourage well-informed and healthier food choices. Thus, FOPWLs can promote health equity by empowering all consumers to identify foods high in critical nutrients and discouraging their consumption [6]. A collection of studies has indicated that FOPWLs have a supporting effect on lowering perceived healthfulness of products high in the abovementioned critical nutrients [7–9]. Packer et al. reported that among a large representative British sample, the FOPWLs were effective in improving participants’ ability to correctly rank products according to healthfulness [7]. In Chile, it has been shown that FOPWLs are used as an informative tool for mothers to determine the healthfulness of products while shopping for their families [10]. Roberto et al. and Arrúa et al. highlighted that among other nutrition labeling schemes, the FOPWL system improved consumer ability to identify products high in critical nutrients [8,9].

Moreover, in Mexico, before the FOPWL policy, an excise tax of one peso per liter was implemented in 2014 on sugar-sweetened beverages (SSBs), which may have incentivized brands to reformulate their products [11]. After the SSB tax was imposed, SSB purchases decreased and water purchases increased, with the greatest change in magnitude seen among those in lower-income and urban households [12]. Currently, there is no data regarding product reformulation in Mexico once the tax or the warning for NNS were implemented. However, countries such as Chile, Peru, and Uruguay, where the warning for NNS was not included, saw product reformulation where added sugars were partly replaced with NNS [5].

NNS also termed artificial sweeteners and non-caloric sweeteners are a class of food additives that are used to sweeten foods and beverages without providing energy in the form of calories [13]. The consumption of NNS in processed foods and beverages has rapidly been increasing in Mexico [13]. Among four countries, including the US, New Zealand, Mexico, and Australia, Mexico was reported to have the highest prevalence of NNS in their processed foods and beverages (11%) according to data collected in 2015–2016 [14].

Evidence regarding the long-term effects of NNS consumption remains inconclusive, particularly for children’s use [15–20]. Recently, the World Health Organization (WHO) issued a conditional recommendation, based on low- certainty evidence, against the use of NNS to control long-term body weight or reduce risk of NCDs in adults and children [16,20]. The WHO’s guideline was largely influenced by prospective observational studies that suggested a higher NNS intake was associated with increased BMI, obesity, type 2 diabetes, cardiovascular disease, and cardiovascular mortality, despite short-term randomized controlled trials (RCTs), showing reductions in body weight and BMI with NNS consumption [16]. Notably, the WHO prioritized observational data due to the perceived limitations of RCTs in demonstrating long-term impacts, which contributed to the conditional nature of their recommendation. In contrast, a more robust analysis was conducted by Lee and colleagues, who employed changes in exposure (rather than baseline or prevalent exposure) and modeled intended substitution to better address the limitations of traditional observational studies​, such as reverse causality and residual confounding. By focusing on substitution effects and controlling for baseline adiposity, Lee et al. concluded that NNS consumption, when replacing SSBs, was associated with lower body weight, a reduced risk of obesity, and favorable cardiometabolic outcomes, such as a decreased risk of coronary heart disease and cardiovascular mortality [18]. However, research on NNS consumption in children remains limited.

FOPWL implementation has been associated with a reduction in the perceived healthfulness of products labeled as “high in” or “excess” of critical nutrients [7–9,21], however, evidence about the disclosure of NNS presence is limited. Arellano-Gómez et al. examined the impact of the NNS cautionary legend of the Mexican FOPWLs on perceived healthfulness of beverages aimed for children [22]. The study found that among adults and youth (10–17 y), the NNS legend decreased perceived healthfulness [22]. Building up on this study, our objective was to compare the impact of the NNS cautionary legend with the “excess sugars” warning label on parents’ preference and perceived healthfulness of fruit-based beverages aimed for children; this mirrors a real-life scenario where consumers must decide between a sugar sweetened beverage containing NNS or a beverage excessive in added sugars. We also aimed to test if parents’ perceptions and preferences differed by nutrition knowledge and previous knowledge on NNS.

## Materials and methods

### Data collection and study design

Data from both the first and third waves of the EPHA niñ@s (Study of the Perception and Dietary Habits in Children, for its acronym in Spanish) cohort collected by the Mexican National Institute of Public Health (INSP-Spanish acronym) was used for the data analysis. EPHA niñ@s cohort was an online cohort of Mexican children aged 5–10 years and either a parent or other primary caregiver across 32 states of Mexico. The aim of the cohort was to assess parents’ and children’s food perceptions and preferences and children’s dietary intake over time. The first wave was conducted from November 14^th^ 2020 to April 27^th^ 2021, and the third wave was conducted from July 21^st^ to November 19^th^ 2022. Data from the second wave was disregarded because it was not relevant for the purposes of this study.

Participants were recruited from paid advertisements from the social media accounts of the study. All participants had internet access at home, a child between 5–10 years with no major chronic conditions, eating disorders, or food allergies, permanent residence in Mexico for the duration of the study, and no current employment within the food industry. Participants that met the abovementioned inclusion criteria, were invited to participate in the study. Written informed consent was requested to all adults. As an incentive, they were given a gift card on completion and participated in raffles of electronic tablets. Participants completed a self-administered questionnaire on their personal devices, through a personalized link that was sent to their email, which could be accessed in several sessions for continual saving until completion. For the analysis, all parents or caregivers that completed the online questionaries for both the first and third waves were included (n = 844).

The study received approval from the Research and Ethics Committees from National Institute of Public Health. Participants provided written consent by reading the informed consent letter and selecting the option that they agreed to participate in the study.

### Preference and healthfulness perception

**Fig 2:**
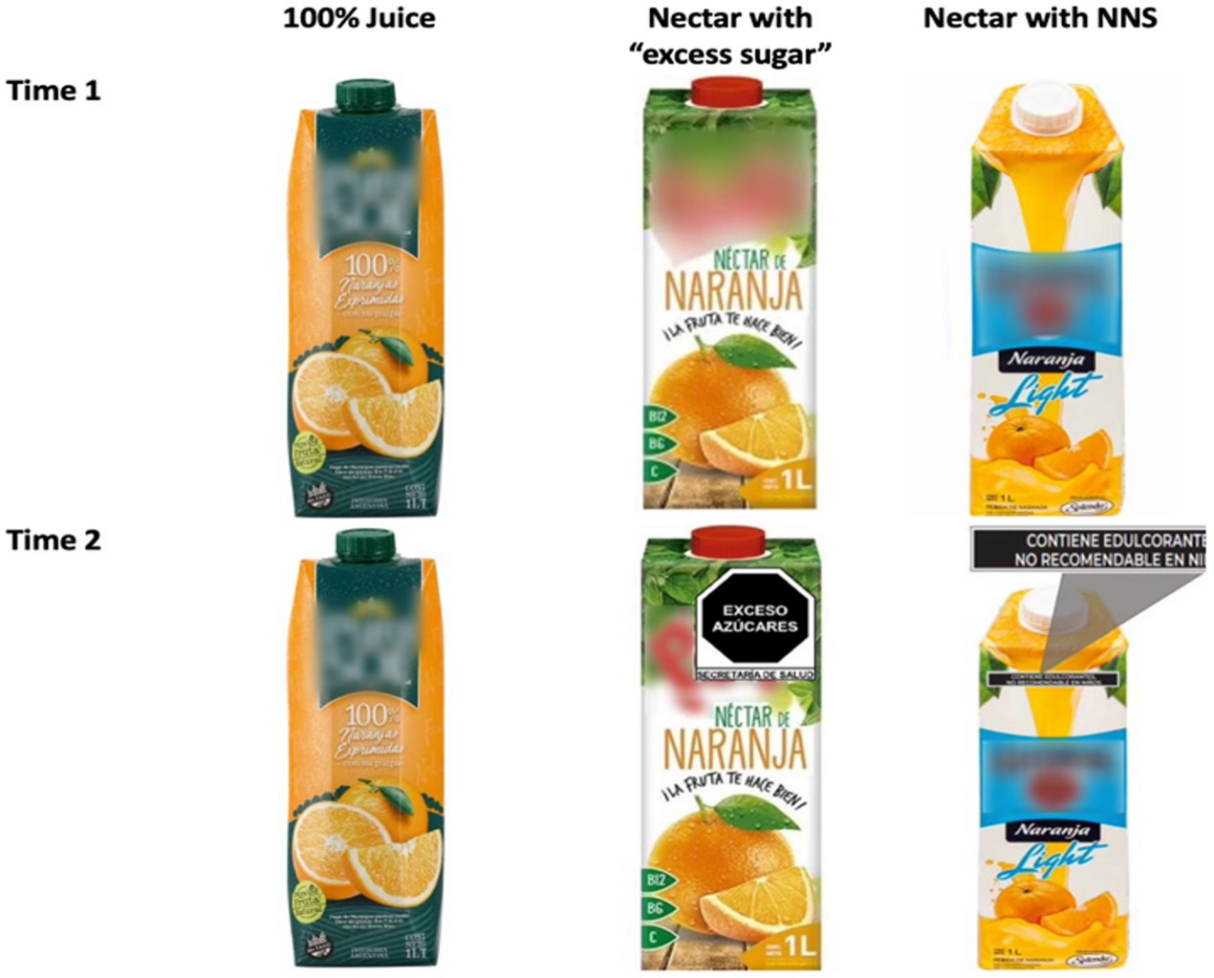
Images with FOPWL by time of data collection.

A section of the online questionnaire consisted of an experiment to test the effect of the FOPWL on preferences and perceived healthfulness of specific examples of food and beverage packaged products. During wave 1, products were presented without FOPWL and in wave 3, the tested products were displayed with the FOPWL. The experiment tested several conditions in different food categories (e.g. ready-to-eat cereals, juices and fruit beverages, and yogurt). The condition related to NNS was tested in the fruit-based beverages, and thus for the purposes of this study, only the analysis from the fruit-based beverage category is presented. The options on this category included a 100% fruit juice, a nectar with excess sugars, and a nectar with NNS marketed as “light”. The first wave of the study represented the baseline condition in which all participants saw images of the front panel of products without FOPWLs. In the third wave of the study, the same questions were asked, but with images of the front panel of products with the corresponding FOPWLs. Specifically, the 100% juice did not have any FOPWL, the nectar with sugar content 10% or more of the total calories had the “excess sugars” label, and the nectar with NNS had the precautionary legend of “contains non-nutritive sweeteners, avoid in children” (Fig 2). The labels were enlarged for the experiment so that they were fully visible in the online questionnaire. To avoid brand recognition and pre-established preferences, the products presented were sourced from other Latin American countries, and thus not found available in Mexican grocery stores. From this point onwards, the first and third waves are referred to as time 1 and time 2, respectively.

To assess preference, parents were asked, *“Which one would you prefer for your son or daughter*?*”*, having to select between 100% fruit juice or nectar with “excess sugars.” Subsequently, parents were presented with nectar containing excess sugars versus nectar with NNS. To assess healthfulness perception, parents were asked *“how healthy do you find this juice*?*”* for each of the three products with response options of 1) not at all healthy, 2) not very healthy, 3) somewhat healthy, and 4) very healthy.

### Covariates

Age, sex, marital status, education level, nutrition knowledge, and the parent’s previous knowledge of what NNS are, as well as the geographic region and socioeconomic status (SES) of the household were obtained for all participants. Education was classified as less than or equal to incomplete high school, complete high school, or incomplete college degree, and greater than or equal to college degree.

Knowledge of nutrition was assessed by the total points given based on the answer choice of the parents for a set of 11 foods, including six healthy foods (mango, carrot, banana, zucchini, beans, and corn tortilla) and five less healthy foods (flour tortilla, turkey sausage, cereal bar, chocolate milk, and cured ham). Response options ranged from strongly agree to strongly disagree, with five options. For healthy foods, 5 points were given if “strongly agree” was selected, 2.5 for “agree”, 0 for nor agree/nor disagree, -2.5 for “disagree”, and -5 for “strongly disagree”. The points were reversed for less healthy points, as such -5 was given for “strongly agree” to 5 for “strongly disagree”. The total points were added and organized into tertiles (high, medium, and low nutrition knowledge). Previous knowledge of NNS was assessed with the question *“do you know what non-nutritive sweeteners are*?*”* (response options: yes or no).

The geographic area was divided into four regions: North, Center, Mexico City, and South. The household SES was assessed using the AMAI 2022 socio-economic levels index (Mexican Association of marketing Research and Public Opinion Agencies, for its acronym in Spanish) [23]. The index classifies the households into seven levels (from higher to lower: A/B, C+, C, C-, D+, D, E) based on the total collection of points gathered from the following variables: level of education of the head of the household, number of bathrooms, number of vehicles, household internet access, number of household members age 14+ who are employed, and number of bedrooms.

### Statistical analysis

Descriptive statistics were estimated to present the sample’s characteristics. To assess the influence of FOPWLs over time, including the precautionary legend for NNS, on preference and healthfulness perception for fruit-based beverage products we used generalized linear models (GLM) with identity link and binomial family functions to estimate proportion differences. We fitted one model for each preference test. Healthfulness perception was dichotomized as healthy (response options: somewhat healthy and very healthy) and not healthy (response options: not at all healthy and not very healthy), and we used one model for each product. All GLMs were adjusted for household SES, nutrition knowledge and previous knowledge of NNS at baseline, and accounted for repeated measures using cluster intragroup correlation. To further assess if parents’ preferences and healthfulness perception differed by nutrition knowledge and previous knowledge of NNS, we used the same models previously described, but included an interaction term of time with preference and healthfulness perception.

Stata’s margins command was used to estimate the average predicted proportions. A significance level of 5% (p value<0.05) was used as the criterion for main analyses, and a level of 10% for interactions. All statistical analyses were performed using STATA 15 (Stata Corp, College Station, TX).

## Results

We analyzed data from 844 adult parents (ages ranging 18–59 years). Most sample participants were female (93.6%) and living at the Center of the country (37.3%). Most of the sample consisted of individuals who were either were married or living together (70.6%). Almost half of the parents (45.5%) had at least a college degree. The sample had 24.5% of participants belonging to lower SES, 56.7% to middle SES, and 18.7% to higher SES. Most of the parents reported knowing what NNS are (80.7%). The participants’ knowledge of nutrition was classified as 39.8% with low knowledge, 28.3% with medium knowledge, and 31.9% with high knowledge (Table 1).

**Table 1:** Parents’ characteristics (n = 844) for the EPHA niñ@s cohort.

	N	%
Age (years): median [range]	34 [18–59]	
Gender		
Female	790	93.6
Male	52	6.2
Prefer not to answer	2	0.2
Geographic Area		
North	162	19.2
Center	315	37.3
Mexico City	103	12.2
South	264	31.3
Marital Status		
Single	103	15.9
Married/Cohabitant	655	70.6
Separated/Divorced/Widow	86	13.5
BMI		
Underweight/normal weight	251	29.7
Overweight	224	26.5
Obesity	123	14.6
Did not answer/did not know weight or height	246	29.2
Education		
Less than or incomplete high-school	200	23.7
Complete high-school and incomplete college degree	260	30.8
College degree or greater	384	45.5
Socioeconomic Status (SES)		
D and D+ (lower)	207	24.5
C-, C and C+ (middle)	479	56.8
A/B (higher)	158	18.7
Knowledge of Non-Nutritive Sweeteners		
Yes	681	80.7
No	163	19.3
Knowledge of Nutrition		
Low knowledge	336	39.8
Medium knowledge	239	28.3
High knowledge	269	31.9

### Parental preferences for fruit-based beverage products

At time 1 (no FOPWL), when asked to choose between 100% juice and nectar with "excess sugars," 72.3% of parents preferred juice, and 27.7% preferred nectar with "excess sugars" (Table 2). At time 2 (with FOPWL), 84.8% of parents preferred juice, while 15.2% preferred nectar with "excess sugars". Thus, at time 2, 12.5% fewer parents preferred nectar with the warning label of "excess sugars" compared to 100% fruit juice (p<0.001).

**Table 2:** Parental preferences for fruit-based beverage products with and without FOPWL*.

	Percentage	95%CI	p-value
First test: Preferred nectar with “excess sugars” over 100% juice			
Time 1**	27.7	[24.7, 30.6]	
Time 2	15.2	[12.9, 17.6]	
Difference	-12.4	[-16.0, -8.8]	p<0.001
Second test: Preferred nectar with NNS over nectar with “excess sugars”			
Time 1	33.9	[30.7, 37.1]	
Time 2	12.1	[9.9, 14.3]	
Difference	-21.8	[-25.5, -18.0]	p<0.001

Abbreviations: CI, confidence interval; NNS, non-nutritive sweeteners; pp, percentage points.

*Difference estimated from generalized linear models, adjusted for SES, parents’ knowledge of nutrition and previous knowledge of NNS at baseline. Time 1 and time 2 values obtained from Stata’s -margins- command.

**Time 1, no warning labels; time 2, warning label of “excess sugars” for nectar and precautionary legend of “contains non-nutritive sweeteners, avoid children” for nectar with NNS.

Similarly, when asked to choose between nectar with "excess sugars" and nectar with NNS, 66.1% of parents preferred nectar with "excess sugars," and 33.9% preferred nectar with NNS at time 1 (no FOPWL). At time 2 (with FOPWL), 87.9% preferred nectar with "excess sugars," and 12.1% preferred nectar with NNS. Therefore, after the introduction of the “contains NNS, avoid in children” legend in time 2, 21.8% fewer parents preferred nectar with NNS over nectar with “excess sugars”(p<0.001).

**Fig 3:**
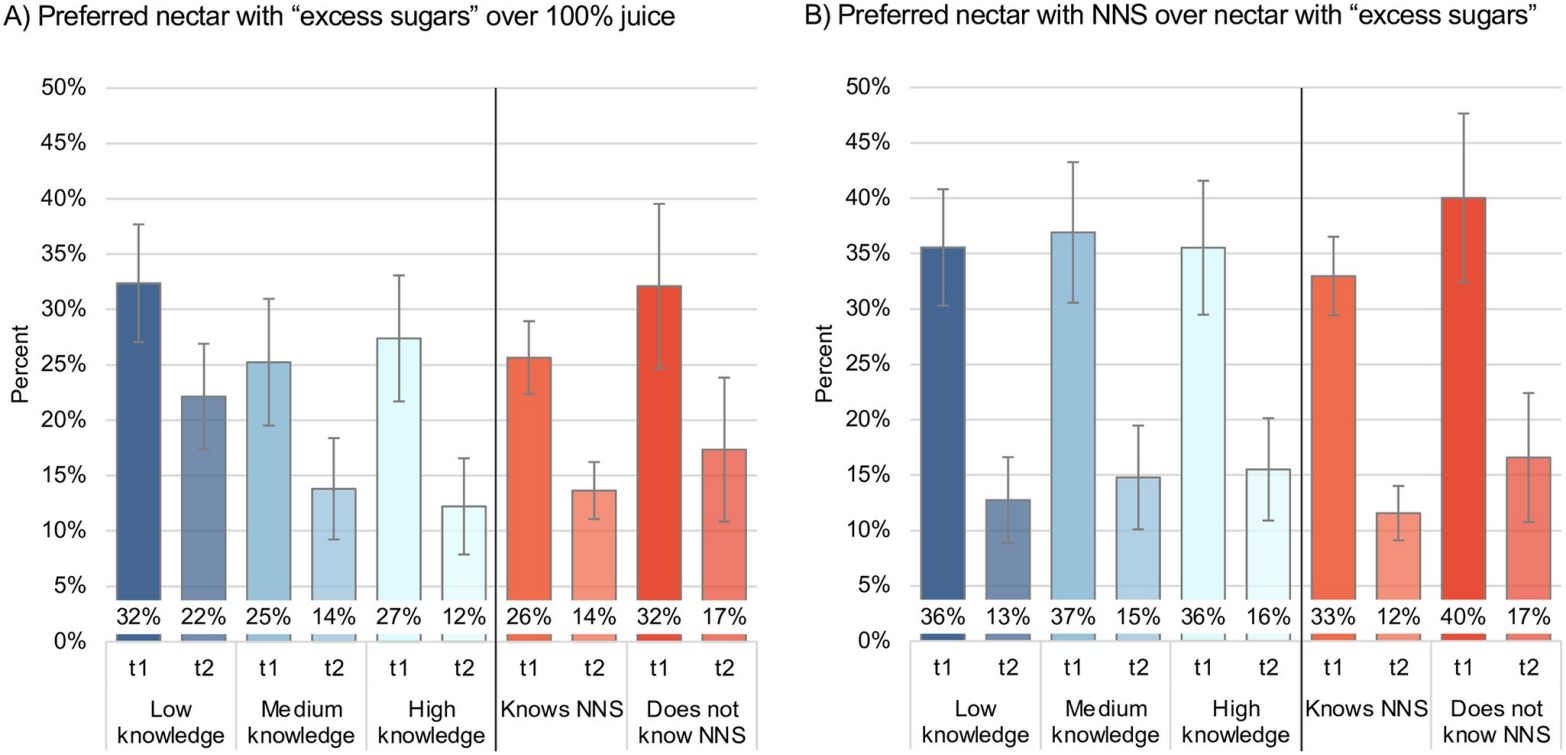
Parental preferences for fruit-based beverage products by nutrition knowledge and previous knowledge of NNS. Abbreviations: NNS, non-nutritive sweeteners, t1, time 1; t2, time 2. * Estimates from generalized linear models. Model for nutrition knowledge includes interaction by time, and adjusted by SES and previous knowledge of NNS at baseline. Model for previous knowledge of NNS includes interaction by time, and adjusted by SES and nutrition knowledge at baseline. Time 1 and time 2 values obtained from Stata’s -margins- command. **Time 1, no warning labels; time 2, warning label of “excess sugar” for nectar and precautionary legend of “contains non-nutritive sweeteners, avoid in children” for nectar with NNS.

Preference results did not differ by nutrition knowledge nor by previous knowledge of NNS (p>0.10) (Fig 3).

### Parental healthfulness perception for fruit-based beverage products

At time 1, 58.8% of parents perceived the beverage without warning labels (100% fruit juice) as healthy, and increased to 76.7% at time 2 (Difference: 17.9 percentage points (p<0.001) (Table 3). On the contrary, 35.1% of parents perceived the nectar with “excess sugars” as healthy and decreased to 25.4% at time 2 (Difference = -9.8 percentage points, p<0.001). Finally, 35.9% of parents perceived the nectar with NNS as healthy at time 1 and decreased to 14.3% at time 2 (Difference: -21.6 percentage points, p<0.001).

**Table 3:** Percentage of parents that perceived fruit-based beverage products as healthy with and without FOPWL*.

Fruit-Based Beverage Product	Percentage	95%CI	p-value
100% Fruit Juice			
Time 1**	58.8	[55.5, 62.1]	
Time 2	76.7	[74.0, 79.5]	
Difference	17.9	[14.1, 21.7]	p<0.001
Nectar with “excess sugars”			
Time 1	35.1	[32, 38.3]	
Time 2	25.4	[22.6, 28.1]	
Difference	-9.8	[-13.5, -6]	p<0.001
Nectar with NNS			
Time 1	35.9	[32.6, 39.2]	
Time 2	14.3	[12, 16.6]	
Difference	-21.6	[-25.5, -17.7]	p<0.001

Abbreviations: CI, confidence interval; NNS, non-nutritive sweeteners.

*Difference estimated from generalized linear models, adjusted for SES, parents’ knowledge of nutrition and previous knowledge of NNS at baseline. Time 1 and time 2 values obtained from Stata’s -margins- command.

**Time 1, no warning labels; time 2, warning label of “excess sugar” for nectar and precautionary legend of “contains non-nutritive sweeteners, avoid in children” for nectar with NNS.

**Fig 4:**
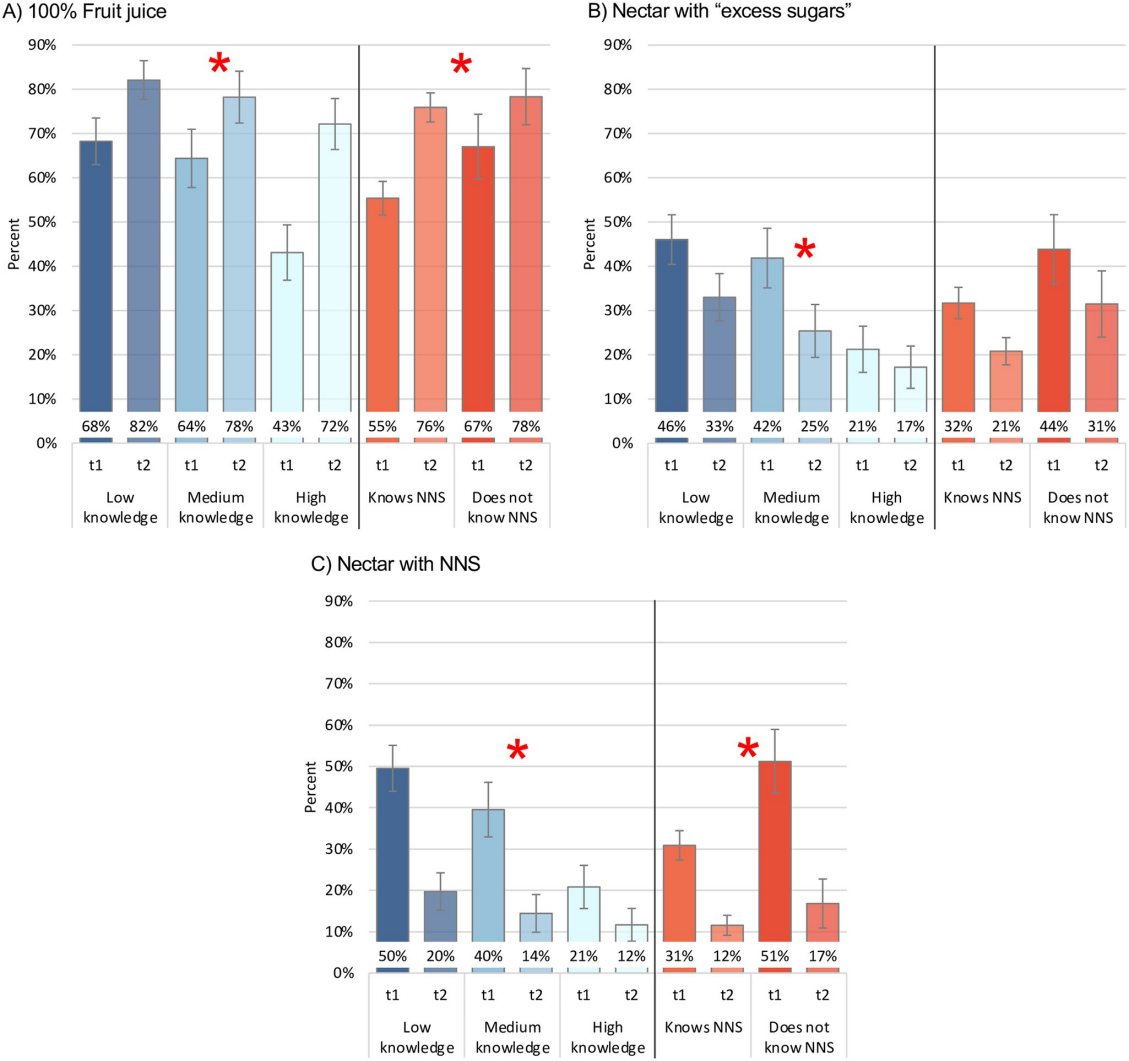
Percent of parents that perceived fruit-based beverage products as healthy by nutrition knowledge and previous knowledge of NNS. Abbreviations: NNS, non-nutritive sweeteners, t1, time 1; t2, time 2. * Estimates from generalized linear models. Model for nutrition knowledge includes interaction by time, and adjusted by SES and previous knowledge of NNS at baseline. Model for previous knowledge of NNS includes interaction by time, and adjusted by SES and nutrition knowledge at baseline. Time 1 and time 2 values obtained from Stata’s -margins- command. Red star indicates p<0.1 for interactions. **Time 1, no warning labels; time 2, warning label of “excess sugar” for nectar and precautionary legend of “contains non-nutritive sweeteners, avoid in children” for nectar with NNS.

Fig 4 shows the percentage of parents that perceived fruit-based beverage products as healthy by nutrition knowledge and previous knowledge of NNS. For 100% juice, the largest increase in percentage points was seen among parents with higher nutrition knowledge and previous knowledge of NNS, whereas the percentages among those with low and medium nutrition knowledge and no previous knowledge of NNS were high since time 1 (p for interaction <0.10). On the contrary, the impact of the FOPWL on nectars with “excess sugar” and the cautionary legend on NNS had a notably greater impact among parents with low and medium nutrition knowledge (for both nectars) and with no previous knowledge of NNS (only for nectar with NNS). While a higher proportion of parents with low and medium nutrition knowledge perceived both nectars as healthy at time 1, the percentages decreased markedly with the inclusion of the FOPWL and the cautionary legend on NNS at time 2 (p for interaction <0.10).

## Discussion

The results presented in this study show that the fruit-beverage with “excess sugars” warning label or with the NNS cautionary legend of the Mexican FOPWL, had a lowering effect on preferences and perceived healthfulness of parents of school-aged children. Also, we examined pre-existing factors at the consumer level such as previous nutrition knowledge and prior knowledge of NNS, as these factors may impact the influence of the cautionary legend and FOPWL. Our results showed that the FOPWL and the cautionary legend had a higher effect on those with low and middle nutrition knowledge and no prior knowledge of NNS on perceived healthfulness of nectars.

Our results are consistent with other studies that have investigated the impact of FOPWL lowering the effect of perceived healthfulness of products that contain excessive critical nutrients [7–9] and NNS [22]. Our findings show that the inclusion of cautionary legend on NNS led parents to perceive the fruit-based product with the cautionary legend as less healthy than the product with the “excess sugar” warning label. Recently, the WHO released a conditional recommendation advising adults and children not to use NNS to control long-term body weight or reduce risk of NCDs [16]. This recommendation was based on low-certainty evidence, largely from observational studies that suggested higher NSS intake was associated with increased BMI, obesity, type 2 diabetes, and cardiovascular disease [16,20,24]. Moreover, there is some concern based on evidence that NSS may increase the desire for sweet tastes, potentially leading to greater calorie consumption [25]. Yet, analysis of prospective studies using change in exposure assessments and substitution analysis showed that NNS consumption when replacing SSBs was associated with lower body weight, a reduced risk of obesity, and favorable cardiometabolic outcomes [18]. However, there is still a scarcity of robust scientific evidence specifically related to long-term intake of NNS in children. Even so, parents perceived the fruit-based product with the cautionary legend as less healthy than the product with the “excess sugar” warning label regardless of previous knowledge of NNS.

For the first time, our study also reported the influence of the NNS cautionary legend on preference and healthfulness perception compared to the “excess sugar” warning label, a fact faced by consumers when selecting sweetened beverages. Our results indicate that the cautionary legend has a greater impact on preference and on healthfulness perception of fruit-based beverage products compared to the “excess sugar” warning label. The addition of the cautionary legend may be more effective in helping parents correctly identify products with NNS for their children.

Regulation of the Mexican FOPWL system specifies that prepacked foods and non-alcoholic beverages must display the FOPWL in a clear format that can truthfully warn consumers about the content of critical nutrients and ingredients that pose risks to their health if consumed excessively [26]. Both the FOPWL and cautionary legend are in a simple and clear format to help consumers identify products containing excessive content of calories and critical nutrients, along with products with any amount of added caffeine or NNS. This may influence how easily consumers interpret the information when evaluating these products, which may be relevant during moments of excessive stimuli and distractions when making purchasing decisions [21]. Further, it enables consumers to condense information by considering the presence or absence of warning labels when deciding to purchase or refrain from buying the products [21]. Warning labeling therefore has an equity impact by providing the knowledge and information to all consumers to make informed choices about the food and beverage products they purchase. Our results align with this, as the “excess sugars” warning label and the cautionary legend on NNS supported a larger decrease for healthfulness perception among parents with low and medium nutrition knowledge compared to parents with high nutrition knowledge. Including the cautionary legend on NNS may expand the knowledge of consumers by providing additional nutrition information in an understandable format so that they may opt for the least harmful food and beverage product according to the Mexican FOPWL system.

In contrast, the exclusion of the cautionary legend on NNS may deny transparency by not offering consumers a complete view to make informed purchasing decisions. Chile was the first country to implement the mandatory FOPWL, consisting of a black octagon, including the words “high in” calories, sugars, saturated fats, and/or sodium [27]. In their regulation, they could not implement the cautionary legend on NNS. Consequently, products were reformulated to substitute partially or totally the added sugars with NNS by the food and beverage industry [28]. This may have been in response to the requirement under the Chilean law to enforce labels indicating “high in sugars” and banning the sale of products “high in sugars” in schools. Results from a study showed that in Chile, there were significant increases in purchases of beverages containing NNS, decreases in beverages containing only caloric sweeteners, and minimal differences among foods [29]. An additional study found that overall, the number of products with NNS in Chile has been increasing since the implementation of the law [30]. Furthermore, daily consumption of NNS may exceed the acceptable daily intake for children when considering the high number of food and beverage products with NNS that are specifically oriented to children [30]. Therefore, after learning about Chile’s experience, Mexico and later Argentina included a cautionary legend on NNS in their labeling law [22]. This study supports the inclusion of the cautionary legend, as our results indicate an influence of the cautionary legend on healthfulness perception and preference for different fruit-based beverage products. Yet, our study did not measure whether the inclusion of the cautionary legend on NNS will decrease purchases of products containing NNS. In addition, consumers should have full transparency regarding the presence of NNS in products when making purchasing decisions for themselves and for their families [28].

The study had some limitations. The sample consisted of mostly highly educated parents and cannot be generalized to the Mexican population. Additionally, the cohort received an online survey where pictures of three different fruit-based beverage products were presented to investigate perception of healthfulness and preference. The effect may have differed if the parents were presented with these fruit-based beverage products in an actual shopping environment rather than on an electronic screen. Furthermore, the warning label and precautionary legend were enlarged for the experiment. Although this may have drawn more attention to them than the standard-sized labels and may not fully reflect real-world conditions, we chose to make them fully visible because some participants completed the self-administered questionnaire on their cell phones, which could have limited their ability to read the legend.

The study also had several strengths. Data collection occurred after the implementation of the FOPWL in Mexico. This would align the experimental results more closely with real-world conditions, as participants were already familiar with the WL. Moreover, conducting the experiments over waves rather than at a single timepoint enhances the real-life applicability of the results, and probably reduced bias in responses, given that participants are unlikely to recall their answers from previous waves. Other strengths of this study also include using a sample of Mexican parents from all regions of the country.

The FOPWL system and cautionary legend on NNS may not provide a cure-all for the prevention of overweight and obesity, as well as diet related NCDs. However, nutrition labeling can enhance understanding and use of nutrient information on food and beverage products, acting as a component of a comprehensive approach alongside other nutrition policy efforts to address obesity and diet related NCDs. In Mexico, the FOPWL, specifically through the cautionary legend “avoid in children” is implemented for products containing NNS. This places the responsibility on adults to interpret and determine the healthfulness of these products for their own consumption. Future research should investigate how adults perceive the healthfulness of products containing NNS for their own consumption, in context of public health messaging and guidelines. Additionally, studies should explore the actual impact of NNS on purchasing and consumption behaviors among both children and adults. Understanding these behavioral outcomes will be crucial for determining the effectiveness of the FOPWL system in guiding dietary choices.

## Conclusion

Our findings post-implementation of FOPWL in Mexico suggest that when the cautionary legend on NNS and “excess sugars” warning labels are present on fruit-based beverage products the percentage of parents that perceive fruit-based beverage products as healthy significantly decreases. Additionally, when parents are comparing two fruit-based beverage products with the cautionary legend and FOPWL present, more parents perceive the product with the cautionary legend on NNS as less healthy than the product with the FOPWL on excess sugar. Including FOWPL in food and beverage products may help direct parents’ behavior toward a healthier diet encouraging them to consider the nutritional quality of products they choose to buy for the household.

## Supporting information

S1 Dataset (CSV)

## References

[1] FAO, OPS, WFP y UNICEF. Panorama de la seguridad alimentaria y nutrición en América Latina y el Caribe 2019. 2019. https://www.fao.org/fileadmin/user_upload/rlc/docs/panorama2019/Panorama2019.pdf.

[2] PAHO. Ultra-processed food and drink products in Latin America: Trends, Impact on Obesity, Policy Implications. 2015. https://iris.paho.org/bitstream/handle/10665.2/7699/9789275118641_eng.pdf?sequence=5&ua=1.

[3] White M, Barquera S. Mexico Adopts Food Warning Labels, Why Now? Health Syst Reform. 2020;6: e1752063. doi: 10.1080/23288604.2020.1752063 32486930

[4] PAHO. Pan American Health Organization Nutrient Profile Model. Washington, D.C; 2016. https://iris.paho.org/bitstream/handle/10665.2/18621/9789275118733_eng.pdf.

[5] Contreras-Manzano A, Cruz-Casarrubias C, Munguía A, Jáuregui A, Vargas-Meza J, Nieto C, et al. Evaluation of the Mexican warning label nutrient profile on food products marketed in Mexico in 2016 and 2017: A cross-sectional analysis. Piernas C, editor. PLOS Med. 2022;19: e1003968. doi: 10.1371/journal.pmed.1003968 35442949 PMC9067899

[6] Pettigrew S, Jongenelis MI, Hercberg S, Julia C. Front-of-pack nutrition labels: an equitable public health intervention. Eur J Clin Nutr. 2023;77: 135–137. doi: 10.1038/s41430-022-01205-3 36085363 PMC9876787

[7] Packer J, Russell SJ, Ridout D, Hope S, Conolly A, Jessop C, et al. Assessing the Effectiveness of Front of Pack Labels: Findings from an Online Randomised-Controlled Experiment in a Representative British Sample. Nutrients. 2021;13: 900. doi: 10.3390/nu13030900 33802115 PMC7999818

[8] Roberto CA, Ng SW, Ganderats-Fuentes M, Hammond D, Barquera S, Jauregui A, et al. The Influence of Front-of-Package Nutrition Labeling on Consumer Behavior and Product Reformulation. Annu Rev Nutr. 2021;41: 529–550. doi: 10.1146/annurev-nutr-111120-094932 34339293

[9] Arrúa A, Machín L, Curutchet MR, Martínez J, Antúnez L, Alcaire F, et al. Warnings as a directive front-of-pack nutrition labelling scheme: comparison with the Guideline Daily Amount and traffic-light systems. Public Health Nutr. 2017;20: 2308–2317. doi: 10.1017/S1368980017000866 28625228 PMC10262271

[10] Correa T, Fierro C, Reyes M, Dillman Carpentier FR, Taillie LS, Corvalan C. “Responses to the Chilean law of food labeling and advertising: exploring knowledge, perceptions and behaviors of mothers of young children.” Int J Behav Nutr Phys Act. 2019;16: 21. doi: 10.1186/s12966-019-0781-x 30760273 PMC6375144

[11] Mordor Intelligence. MEXICO FOOD SWEETENER MARKET SIZE & SHARE ANALYSIS—GROWTH TRENDS & FORECASTS (2023–2028). 2023. https://www.mordorintelligence.com/industry-reports/mexico-food-sweetener-market.

[12] Colchero MA, Molina M, Guerrero-López CM. After Mexico Implemented a Tax, Purchases of Sugar-Sweetened Beverages Decreased and Water Increased: Difference by Place of Residence, Household Composition, and Income Level. J Nutr. 2017;147: 1552–1557. doi: 10.3945/jn.117.251892 28615377 PMC5525113

[13] Romo-Romo A, Brito-Córdova GX, Aguilar-Salinas CA, Cano-García de León C, Farías-Name DE, Reyes-Lara L, et al. Beliefs concerning non-nutritive sweeteners consumption in consumers, non-consumers, and health professionals: a comparative cross-sectional study. Nutr Hosp. 2022 [cited 18 Jul 2023]. doi: 10.20960/nh.04046 36094057

[14] Dunford E, Taillie L, Miles D, Eyles H, Tolentino-Mayo L, Ng S. Non-Nutritive Sweeteners in the Packaged Food Supply—An Assessment across 4 Countries. Nutrients. 2018;10: 257. doi: 10.3390/nu10020257 29495259 PMC5852833

[15] Debras C, Chazelas E, Sellem L, Porcher R, Druesne-Pecollo N, Esseddik Y, et al. Artificial sweeteners and risk of cardiovascular diseases: results from the prospective NutriNet-Santé cohort. BMJ. 2022; e071204. doi: 10.1136/bmj-2022-071204 36638072 PMC9449855

[16] World Health Organization (WHO). Use of non-sugar sweeteners: WHO guideline. 2023. file:///Users/claudiacalderonkristen/Downloads/9789240073616-eng%20(1).pdf. 37256996

[17] Swithers SE. Artificial sweeteners are not the answer to childhood obesity. Appetite. 2015;93: 85–90. doi: 10.1016/j.appet.2015.03.027 25828597

[18] Lee JJ, Khan TA, McGlynn N, Malik VS, Hill JO, Leiter LA, et al. Relation of change or substitution of low-and no-calorie sweetened beverages with cardiometabolic outcomes: a systematic review and meta-analysis of prospective cohort studies. Diabetes Care. 2022;45: 1917–1930. doi: 10.2337/dc21-2130 35901272 PMC9346984

[19] McGlynn ND, Khan TA, Wang L, Zhang R, Chiavaroli L, Au-Yeung F, et al. Association of low-and no-calorie sweetened beverages as a replacement for sugar-sweetened beverages with body weight and cardiometabolic risk: a systematic review and meta-analysis. JAMA Netw Open. 2022;5: e222092–e222092. doi: 10.1001/jamanetworkopen.2022.2092 35285920 PMC9907347

[20] Hedrick VE, Nieto C, Grilo MF, Sylvetsky AC. Non-sugar sweeteners: helpful or harmful? The challenge of developing intake recommendations with the available research. bmj. 2023;383. doi: 10.1136/bmj-2023-075293 37813435 PMC11426959

[21] Taillie LS, Hall MG, Popkin BM, Ng SW, Murukutla N. Experimental Studies of Front-of-Package Nutrient Warning Labels on Sugar-Sweetened Beverages and Ultra-Processed Foods: A Scoping Review. Nutrients. 2020;12: 569. doi: 10.3390/nu12020569 32098363 PMC7071470

[22] Arellano-Gómez LP, Jáuregui A, Nieto C, Contreras-Manzano A, Quevedo KL, White CM, et al. Effects of front-of-package caffeine and sweetener disclaimers in Mexico: cross-sectional results from the 2020 International Food Policy Study. Public Health Nutr. 2023;26: 3278–3290. doi: 10.1017/S1368980023002100 37781769 PMC10755452

[23] Nivel socioeconómico AMAI. CUESTIONARIO PARA LA APLICACIÓN DE LA REGLA AMAI 2022 Y TABLA DE CLASIFICACIÓN. 2022. https://www.amai.org/descargas/CUESTIONARIO_AMAI_2022.pdf.

[24] Khan TA, Lee JJ, Ayoub-Charette S, Noronha JC, McGlynn N, Chiavaroli L, et al. WHO guideline on the use of non-sugar sweeteners: a need for reconsideration. Eur J Clin Nutr. 2023;77: 1009–1013. doi: 10.1038/s41430-023-01314-7 37723261 PMC10630128

[25] Qin P, Li Q, Zhao Y, Chen Q, Sun X, Liu Y, et al. Sugar and artificially sweetened beverages and risk of obesity, type 2 diabetes mellitus, hypertension, and all-cause mortality: a dose–response meta-analysis of prospective cohort studies. Eur J Epidemiol. 2020;35: 655–671. doi: 10.1007/s10654-020-00655-y 32529512

[26] PROYECTO de Modificación a la Norma Oficial Mexicana NOM-051-SCFI/SSA1-2010, Especificaciones generales de etiquetado para alimentos y bebidas no alcohólicas preenvasados-Información comercial y sanitaria. Secrretaria de gobernacion; 2019. Available: PROYECTO de Modificación a la Norma Oficial Mexicana NOM-051-SCFI/SSA1-2010, Especificaciones generales de etiquetado para alimentos y bebidas no alcohólicas preenvasados-Información comercial y sanitaria.

[27] Taillie LS, Reyes M, Colchero MA, Popkin B, Corvalán C. An evaluation of Chile’s Law of Food Labeling and Advertising on sugar-sweetened beverage purchases from 2015 to 2017: A before-and-after study. Basu S, editor. PLOS Med. 2020;17: e1003015. doi: 10.1371/journal.pmed.1003015 32045424 PMC7012389

[28] Sylvetsky AC, Rebolledo N, Krieger JW. Nonsugar Sweeteners—Time for Transparency and Caution. JAMA Pediatr. 2024 [cited 24 Feb 2024]. doi: 10.1001/jamapediatrics.2023.6041 38252446

[29] Rebolledo N, Bercholz M, Adair L, Corvalán C, Ng SW, Taillie LS. Sweetener Purchases in Chile before and after Implementing a Policy for Food Labeling, Marketing, and Sales in Schools. Curr Dev Nutr. 2023;7: 100016. doi: 10.1016/j.cdnut.2022.100016 37180088 PMC10111599

[30] Sambra V, López-Arana S, Cáceres P, Abrigo K, Collinao J, Espinoza A, et al. Overuse of Non-caloric Sweeteners in Foods and Beverages in Chile: A Threat to Consumers’ Free Choice? Front Nutr. 2020;7: 68. doi: 10.3389/fnut.2020.00068 32626722 PMC7311776

